# Measuring Gait Quality in Parkinson’s Disease through Real-Time Gait Phase Recognition

**DOI:** 10.3390/s18030919

**Published:** 2018-03-20

**Authors:** Ilaria Mileti, Marco Germanotta, Enrica Di Sipio, Isabella Imbimbo, Alessandra Pacilli, Carmen Erra, Martina Petracca, Stefano Rossi, Zaccaria Del Prete, Anna Rita Bentivoglio, Luca Padua, Eduardo Palermo

**Affiliations:** 1 Department of Mechanical and Aerospace Engineering, Sapienza University of Rome, 00184 Rome, Italy; alessandra.pacilli@uniroma1.it (A.P.); zaccaria.delprete@uniroma1.it (Z.D.P.); 2 Fondazione Don Carlo Gnocchi Onlus, 20121 Milan, Italy; mgermanotta@dongnocchi.it (M.G.); edisipio@dongnocchi.it (E.D.S.); isabellaimbimbo@gmail.com (I.I.); annarita.bentivoglio@policlinicogemelli.it (A.R.B.); luca.padua@unicatt.it (L.P.); 3 Department of Geriatrics, Neurosciences and Orhopaedics, Università Cattolica del Sacro Cuore, 00168 Rome, Italy; carmen.erra@hotmail.it (C.E.); martina.petracca@gmail.com (M.P.); 4 Department of Economics, Engineering, Society and Business Organization (DEIM), University of Tuscia, 01100 Viterbo, Italy; stefano.rossi@unitus.it

**Keywords:** gait quality, gait phases recognition, machine learning, Parkinson’s disease, motor fluctuations, wearable sensor system

## Abstract

Monitoring gait quality in daily activities through wearable sensors has the potential to improve medical assessment in Parkinson’s Disease (PD). In this study, four gait partitioning methods, two based on thresholds and two based on a machine learning approach, considering the four-phase model, were compared. The methods were tested on 26 PD patients, both in OFF and ON levodopa conditions, and 11 healthy subjects, during walking tasks. All subjects were equipped with inertial sensors placed on feet. Force resistive sensors were used to assess reference time sequence of gait phases. Goodness Index (G) was evaluated to assess accuracy in gait phases estimation. A novel synthetic index called Gait Phase Quality Index (GPQI) was proposed for gait quality assessment. Results revealed optimum performance (G < 0.25) for three tested methods and good performance (0.25 < G < 0.70) for one threshold method. The GPQI resulted significantly higher in PD patients than in healthy subjects, showing a moderate correlation with clinical scales score. Furthermore, in patients with severe gait impairment, GPQI was found higher in OFF than in ON state. Our results unveil the possibility of monitoring gait quality in PD through real-time gait partitioning based on wearable sensors.

## 1. Introduction

In the last decade, increasing attention has been paid to novel methodologies for the real-time gait phase detection [1,2]. Assessment of gait temporal parameters has the great potential to objectively evaluate patient recovery during rehabilitation treatments, to discriminate between normal and pathological gait, and to reliably quantify typical hallmarks among gait impairments, such as motor fluctuations in Parkinson’s Disease (PD) [3,4,5,6].

As of today, levodopa is the most effective medication in reducing PD symptoms [7]. However, precisely tailored dosage of levodopa is crucial in limiting side effects, which significantly impinge patients’ capability in performing daily motor activities [8].

Some patients with advanced PD experience motor fluctuations which cause also alternation between higher (ON state) and lower (OFF state) levels of gait quality, due to the long-term levodopa syndrome [8]. Many of these so-called motor fluctuations appear throughout the day, related to drug dosing, worsening functional independence linked to gait quality [9]. Common motor fluctuations are [8]: wearing-off effect, i.e., periods in which patients experience motor impairments earlier the end of dose due to a loss of treatment efficacy, and peak-dyskinesia, i.e., involuntary movements related to a (subjective) over-dose levodopa plasma level. Such motor fluctuations cause a sensible deterioration of patient’s quality of life. Motor fluctuations lead to fatigue and exhaustion, also increasing the risk of fall [10], social withdrawal and isolation and consequent frustration and depression [10].

Currently, patient’s home-diaries are the most adopted method for assessing motor fluctuations, presenting high inaccuracy of patients self-report, especially when OFF and ON episodes closely occur [6]. UPDRS-III rating scale [11] can be used for motor assessment, but it actually limits motor evaluation to clinical setting and expert clinicians. Furthermore, motor and gait assessments are conducted in motion laboratory setting, by using high accurate optical tracking sensors, an expensive methodology that limits the applicability to clinical environments. Recently, the adoption of wearable solutions to assess parkinsonian motor symptoms based on gait segmentation [12] has become prominent. A further developmental stage in these methodologies, aimed at analyzing phases distribution in every single stride, would have a tremendous potential in real-time monitoring of gait disturbance in PD patients. Overcoming limitations of traditional monitoring methods through wearable technologies could provide sensible advantage in drug administration, improving both treatment efficiency, and patient’s quality of life. More specifically, the continuous assessment of gait symptom status during the day on a digital diary, accessible to clinicians, could provide helpful information helping in the periodical adjustment of the dosage for the pharmacological treatment. Furthermore, such a methodology, when coupled with a smartphone app, would have a tremendous potential in advising the patient on his current gait quality, prompting him about risk of fall. Lastly, the measurement could be used to trigger or modulate specific treatments, such as rhythmic auditory stimuli, which proved to improve motor performance [13].

Estimation of gait temporal parameters, such as swing or stride time, allowed the identification of arrhythmic steps leading to falls [14]. Freezing of gait was found to be strictly related to gait asymmetry of swing phase [15]. In general, stance sub-phases, such as loading response, flat-foot and pre-swing, have been considered clinically meaningful parameters to reveal gait pattern changes [4,16,17,18]. In particular, an abnormal loading response leading to instability can be improved with levodopa treatments in PD [19]. Parkinsonian gait is characterized by a different average load distribution with respect to normal one, lower under the heel and higher under midfoot, reflecting the difficulty of patients to produce a normal “heel to toe” roll-over [20]. By increasing midfoot load during flat-foot phase, PD patients compensate possible loss of balance frequently occurring [21]. Furthermore, the reduction of pre-swing phase, caused by decreased plantar forces at forefoot, implies a reduced leg acceleration during swing phase, worsening stride length and gait speed [20]. Thus, daily monitoring of gait phase distribution in loading response, flat-foot, pre-swing, and swing phases, which compose the four-phase model, can play an important role in PD treatment. 

Nowadays several methodologies have been validated to implement the four-phase model, involving different inertial sensor systems and computational algorithms, such as threshold methods and machine-learning approaches [1,16,22,23,24,25,26]. Regarding different sensors’ positions, footwear-based systems are the most used when considering four-phase model [1]. A threshold method, processing pitch angular velocity, the norm of 3D acceleration signal, and the derivative of 3D gyroscope signal norm of foot-worn inertial sensors, was proposed by Mariani et al. to detect swing and stance sub-phases [16]. This method has been validated only on patients before and after surgical treatments for ankle osteoarthritis and aged-matched healthy subjects. Rueterbories et al. proposed a sophisticated threshold-based algorithm for automatic gait event detection by means of the radial and tangential acceleration of the foot [22]. The accuracy of the proposed method has been investigated in healthy adults and in hemiparetic individuals during level walking with a metronome. An ambulatory monitoring system for the estimation of spatial and temporal parameters involving foot sagittal angular velocity was proposed by Sabatini et al. [26]. This approach allowed gait event identification needed for both gait partitioning and strap-down integration. 

A machine-learning approach based on Hidden Markov Model (HMM) was introduced by Mannini et al. for gait segmentation using a foot-mounted gyroscope [23]. Authors validated this methodology both on normal gait and pathological gait, such as Huntington’s disease and post-stroke [27]. Lately, Taborri et al. validated a novel machine-learning approach based on two or more scalar HMM for the control system of a pediatric exoskeleton [28] and the same authors proposed the optimization of the training procedure to avoid the time-consuming subject-specific training procedure [24,25]. These methodologies showed excellent results in comparison to foot-switches as reference system, goodness index was never higher than 0.21 ± 0.17. 

To the best of our knowledge, all the mentioned methods have been tested only on subjects’ population including ankle osteoarthritis, hemiparetics, individuals with Huntington’s disease, cerebral palsy and post-stroke, other than healthy adults. The lack of validation approaches involving parkinsonian both in ON and OFF state may limit the applicability in real-time of these methods due to the unforeseeable gait motor fluctuation levodopa-induced. Therefore, a comparative analysis of different gait partitioning methods for the four-gait model is required to verify the applicability of these methodologies both in ON and OFF levodopa conditions and to assess the best solution in term of accuracy in PD. 

To quantify gait quality fluctuations in PD through gait partitioning, the introduction of synthetic indices based on measured gait phases is crucial. Several examples of overall indices have been proposed in literature to quantify severity of walking condition [29,30,31,32,33,34]. A set of these indices involves kinematic variables that cannot be evaluated through foot-worn sensor systems [29,30,32]. Contrarily, the Gait Variability Index (GVI) relies on spatiotemporal parameters measurable through a footwear-based method [33]. However, Rennie et al. [35] discouraged GVI application in PD due to its low sensitivity in discriminating between PD and healthy patterns. The above descripted parkinsonian gait characteristics, encourage monitoring of the four gait phases distribution, with the aim of preventing falls, detecting postural instability, and assessing motor fluctuations between OFF and ON state. However, the lack of a synthetic index involving the assessment of all the stance sub-phases and swing phase, calls for the development and validation of a novel approach to estimate deviation of PD gait phase distribution from normalcy value.

The purpose of this study is twofold: (i) comparing different gait partitioning methodologies involving threshold methods and machine-learning approaches for the estimation of the four gait phases in patients with PD both in ON and OFF levodopa conditions; and (ii) validating a novel synthetic index, the Gait Phases Quality Index (GPQI), for gait quality monitoring through gait partitioning in PD.

## 2. Materials and Methods

### 2.1. Subjects

Twenty-six patients with idiopathic PD (age: 71.6 ± 6.9 years) and eleven age-matched healthy subjects (age: 70.1 ± 7.8 years) were enlisted in this study. More details on patients are reported in Table 1. All participants presented no intellectual deficit and a full verbal comprehension. They did not undergo any orthopedic and neurological surgery in the last three years before the study. Healthy subjects in the control group (CG) did not present any other pathologies influencing their walking capability. Patients with PD were able to walk independently and the inclusion criteria were: (i) diagnosis of PD based on the established criteria [36]; (ii) UPDRS-III scale difference between ON and OFF within the range 20–30% (assessed by an expert neurologist through UPDRS-III score, both in OFF and ON state); and (iii) occurrence of motor fluctuations. Patients were recruited from the outpatients of the Policlinico Gemelli Hospital in Rome. Written consent was obtained from all participants before performing the experimental procedure and the procedure was approved by the Ethics and Medical Board of the Don Carlo Gnocchi Onlus Foundation, Milan, Italy, where the experiment was carried out.

### 2.2. Experimental Setup

**Figure 1:**
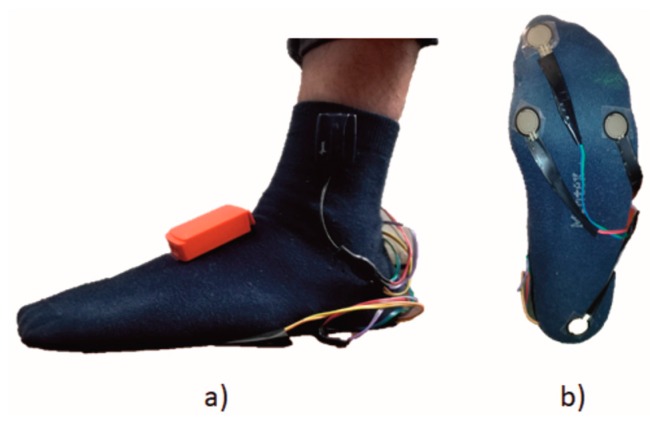
Sensors positioning on subject. (**a**) Position of IMU on participant’s foot; (**b**) position of footswitches under participant’s foot.

All subjects were equipped with two Inertial Measurement Units (IMUs, MTw, Xsens Technologies—Enschede, The Netherlands) attached on the instep of both feet and eight force resistive sensors (Wireless, Wave EMG, Cometa—Milan, Italy), four under each foot, as shown in Figure 1.

Each Inertial Measurement Unit embeds a 3-axes accelerometer (±160 m/s^2^ FS), a 3-axes gyroscope (±1200°/s FS), and a 3-axes magnetometer (±1.5 Gauss FS). Only data from accelerometers and gyroscopes have been used in this study to feed gait-partitioning algorithms. Sensors alignment was performed manually by the same expert operator and each IMU was fixed with elastic straps to limit relative movements between sensor and body segment. IMU sampling rate was set at 50 Hz. Force resistive sensors were used as footswitches (FTSWs) to estimate reference gait events, yielding the reference gait phase sequence. More specifically, four FTSWs were placed under each foot in the following positions: (i) heel; (ii) first metatarsophalangeal; (iii) fifth metatarsophalangeal; and (iv) toe. FTSW data were gathered at 2000 Hz and sent by two wireless modules placed on each foot to the base receiver. FTSWs and IMUs were synchronized in starting and stopping the acquisition. In post-processing, signals from FTSWs and inertial sensors were resampled at 200 Hz. 

### 2.3. Experimental Procedure

All subjects were asked to perform three walking trials along a 20-meters pathway at their comfortable speed. Gait patterns of PD were gathered both in OFF and ON state. OFF state was assessed in early morning after twelve hours of drug free period. Later, ON state was evaluated one hour after the intake of usual morning dopaminergic drug dose. Patients who experienced fatigue were allowed to rest on a chair until they felt ready for the next walking trial. All subjects were able to complete the experimental protocol.

### 2.4. Data Processing and Tested Methods

Data processing and data analysis were performed using MATLAB software (MathWorks, Natick, MA, USA). A gait cycle was assessed as the time period between two consecutive Heel Strikes (HSs). According to the four-phase model, each gait cycle was subdivided into: (i) Loading Response (LR), lasting from Heel Strike (HS) to Toe Strike (TS); (ii) Flat Foot (FF), from Toe Strike to Heel Off (HO); (iii) Pre-Swing (PS), from Heel Off to Toe Off (TO); and Swing (Sw), from Toe Off to next Heel Strike. Reference gait phases were assessed through FTSWs according the following criteria: (i) Loading Response: only FTSW under heel was pressed; (ii) Flat-Foot: all FTSWs were pressed; (iii) Pre-Swing: at least one out of toe, first and fifth metatarsus FTSWs was pressed; (iv) Swing: none of the FTSWs was pressed. The comparative analysis of this study involved four gait-partitioning methods, already introduced in literature for other pathologies, two based on threshold methodologies and two based on machine learning approach. In Table 2, we report a brief description of the four methods. We recommend reading the corresponding papers, cited in the reference section, for a deeper understanding of each algorithm.

The output of all examined methods was the state sequence of LR, FF, PS and Sw. The first three and the last three gait cycles were discarded to avoid gait acceleration and deceleration effects. The accuracy of methods was assessed thought: sensitivity, also expressed as True Positive Rate (TPR); specificity, also expressed as True Negative Rate (TNR); and, the Goodness Index (G), defined as:

(1) $$G=\sqrt{{\left(1-\mathrm{TNR}\right)}^{2}+{\left(1-\mathrm{TPR}\right)}^{2}}$$

where:

(2) $$\mathrm{TPR}=\frac{\mathrm{TP}}{\mathrm{TP}\;+\;\mathrm{FN}}$$

(3) $$\mathrm{TNR}=\frac{\mathrm{TN}}{\mathrm{TN}\;+\;\mathrm{FP}}$$

True Positive (TP) is the number of all transitions rightly detected by each method, compared with transitions obtained from FTSWs. Similarity, True Negative (TN) is the number of all non-transitions rightly detected. Moreover, all transitions and non-transitions wrongly detected were considered as false positive (FP) and false negative (FN), respectively. According to [24], a 60 ms tolerance window was set, centered on each transition state.

Receiver Operating Characteristic (ROC) curve analysis was performed using TPR and TNR. G represents the Euclidean distance between the evaluated point in the ROC space and the point [0, 1], that is the perfect performance in the ROC space, and it ranges into (0, $\sqrt{2}$). Each classifier was considered: (i) optimum when G ≤ 0.25; (ii) good when 0.25 < G ≤ 0.7; (iii) random if G > 0.7, according to [37].

The right and left gait sequences, obtained both for the four methods and FTSWs outputs, were divided in LR, FF, PS and Sw, expressed as a percentage of the gait cycle. To estimate the performance of each algorithm in measuring gait phase percentage, the absolute error for each phase (LR_e_, FF_e_, PS_e_, Sw_e_) was computed as the difference between the percentage of gait phase obtained through each method, and percentage of gait phase obtained through the FTSWs.

### 2.5. Gait Phases Quality Index

In this study we introduced a novel index to synthetically quantify the quality of gait thought gait phases. The Gait Phases Quality Index (GPQI) estimates the difference between the average patient-phase sequence and those of control group. The GPQI was calculated as follows:

(4) $$\mathrm{GPQI}=\sqrt{\overset{2}{\underset{i=1}{\sum }}{\left({\mathrm{LR}}_{\mathrm{PD}}-{\mathrm{mLR}}_{\mathrm{CG}}\right)}^{2}+{\left({\mathrm{FF}}_{\mathrm{PD}}-{\mathrm{mFF}}_{\mathrm{CG}}\right)}^{2}+{\left({\mathrm{PS}}_{\mathrm{PD}}-{\mathrm{mPS}}_{\mathrm{CG}}\right)}^{2}+{\left({\mathrm{Sw}}_{\mathrm{PD}}-{\mathrm{mSw}}_{\mathrm{CG}}\right)}^{2}}$$

where i stands for the side (left and right); LR_PD_, FF_PD_, PS_PD_, Sw_PD_ represent the gait phases percentage of patient with PD and mLR_CG_, mFF_CG_, mPS_CG_, mSw_CG_ are the average values of gait phases in the control group. The GPQI value is the Euclidean distance, in a four-dimensional space of the gait phases’ distribution, between the point determined by the gait phases’ percentages of the examined stride, and the point determined by the average distribution of gait phases among healthy subjects. The GPQI value represents the deviation from healthy gait in terms of gait phases. A GPQI value close to 0% represents a gait pattern similar to the healthy one. Mean GPQI was calculated considering the gait sequence gathered from FTSWs. GPQI was computed both for patients, in OFF and ON state (GPQI^OFF^, GPQI^ON^), to assess levodopa effects on gait phases distribution and for each control-group subject (GPQI^CG^) to quantify how gait patters of healthy subjects differed from the healthy average. Similar to the percentage of gait phases, GPQI was also calculated for all methods, and the absolute error in the estimation of GPQI (GPQI_e_) was computed. More specifically, for each stride, the absolute error was calculated as the difference between GPQI obtained through the FTSWs and GPQI obtained through each one of the gait partitioning methods. All parameters were computed for each walking trial. The average value obtained across the three walking trials was used for the statistical analysis.

### 2.6. Statistical Analysis

Statistical analysis was performed on the following groups: (i) the entire cohort of PD patients (G_0–3_); (ii) a subset of 14 patients (G_0–1_) presenting a mild level of gait impairment; and (iii) a subset of 12 patients (G_2–3_) presenting a severe level of gait impairment. A mild level of gait impairment was addressed when the GAIT sub-item of UPDRS-III value related to OFF condition was lower than 2, otherwise severe level of gait impairment was assigned. Statistical analysis was performed using SPSS package (IBMSPSS Inc., Armonk, NY, USA). All data were tested for normality with the Shapiro-Wilk test. TPR, TNR, G, LR_e_, FF_e_, PS_e_, Sw_e_ and GPQI_e_ values were analyzed using two-way repeated measures ANOVA tests, with Methods (four levels) and Pharmacological Conditions (two levels) as within-subject factors. When significant differences were found, a Bonferroni’s test was performed. The Greenhouse-Geisser correction was adopted when the Mauchly’s test was significant and the assumption of sphericity was violated. Otherwise, *p*-value of sphericity was considered. If the interaction effect Methods × Pharmacological Conditions was significant, the interactions were broke-down, comparing OFF and ON pharmacological conditions separately. More specifically, a one way repeated measures ANOVA with Methods (four levels) was performed both for OFF and ON conditions. Furthermore, a paired *t*-test was computed to assess difference between GPQI^OFF^, GPQI^ON^ and an unpaired *t*-test between GPQI^OFF^, GPQI^CG^ and GPQI^ON^, GPQI^CG^. A ROC curve analysis and the area under the curve (AUC) were used to assess the capability of GPQI to discriminate between OFF and ON state and between normal and pathological gait. Perfect discrimination is related to an area of 100%, while and area of 50% represents a worthless classifier. TPR, TNR, G, LR_e_, FF_e_, PS_e_, Sw_e_ and GPQI_e_ values were analyzed considering the entire PD population (G_0–3_). Conversely, to highlight influence of gait impairment severity, statistical analysis of GPQI were performed considering G_0–3_, G_0–1_ and G_2–3_, separately. Correlations between GPQI values and clinical scale were investigated using Spearman’s Rho analysis on the entire parkinsonian population (G_0–3_,). To evaluate the quality of the linear regression, the correlation coefficient (r) was used. The absolute value of r can be interpreted, in agreement to [38], as: (i) no correlation, if |r| ≤ 0.1; (ii) mild/modest correlation, if 0.1 < |r| ≤ 0.3; (iii) moderate correlation, if 0.3 < |r| ≤ 0.6; (iv) strong correlation, if 0.6 < |r| < 1; and, finally; (v) perfect correlation, if |r| = 1. Furthermore, to evaluate test-retest reliability of GPQI related to the three walking trials, the Intra-class Correlation Coefficients (ICC_3,k_) was calculated on G_0–3_ both in OFF and ON state [39]. Values in range 0.0–0.4 were considered poor, 0.40–0.59 fair, 0.60–0.74 good and 0.75–1.00 to be excellent [40]. Then, to investigate the smallest amount of change in GPQI that unveils a significant gait modification with respect to random measurement error, the Minimal Detectable Change (MDC_95%_) was calculated, in accordance to [41]. The significance level was set at 0.05 for all statistical tests.

## 3. Results

In Table 3, mean and standard deviation of TPR, TNR and G values are reported for all examined methods, for the group G_0–3_. No statistical differences were found between left and right side for TPR, TNR and G values. Therefore, only results from right side were reported.

In OFF state the highest value of TPR and TNR was reached by S-method, HMMsst and HMMspt and the lowest by R-method. In ON state the highest value of TPR was reached by S-method, HMMsst instead the TNR highest value was achieved by S-method, HMMsst and HMMspt. Similarly to the OFF state, the lowest value of TPR and TNR in ON state was reached by R-methods. G value resulted lower than 0.25, for S-method, HMMsst and HMMspt, meaning that optimum performances were achieved both in OFF and ON state by those methods. Only in R-method, G value exceeded the threshold of 0.25, both in OFF and ON state and its performance can be classified in good range (0.25 < G ≤ 0.7). 

Regarding TPR, the two-way repeated measure ANOVA test showed no significant interaction between Pharmacological Condition and Methods (*p* = 0.58). No significant difference was found between OFF and ON state (*p* = 0.08). Furthermore, the main effect Methods resulted significant (*p* < 0.01). In particular, R-method was statistically different from S-method (*p* < 0.01), HHMsst (*p* < 0.01) and HMMspt (*p* < 0.01), as showed by Bonferroni post-hoc test. 

Similar results were obtained for TNR and G value. More specifically, regarding TNR, no statistical interaction between the two main effects was found (*p* = 0.89) and between OFF and ON state (*p* = 0.86). Differences were found between Methods (*p* < 0.01). In particular, R-method was statistical different from S-method (*p* < 0.01), HHMsst (*p* < 0.01) and HMMspt (*p* < 0.01). As regard the G value, no statistical interaction between Pharmacological Condition and Methods was found (*p* = 0.72) and between OFF and ON state (*p* = 0.21). The main effect Methods was significant (*p* < 0.01) with R-method statistically different from S-method (*p* < 0.01), HHMsst (*p* < 0.01) and HMMspt (*p* < 0.01).

In Table 4, mean and standard deviation of Absolute Error of each right gait phase and Absolute Error of GPQI for all methods are reported considering G_0–3_ group. Similarly to TPR, TNR and G value, no statistical difference was found between sides.

Confirming results from G, TPR and TNR, the lowest values of absolute error in the assessment of gait phases percentage was achieved by S-method, HMMsst, and HMMspt. In particular, for R-method the high value of G both in OFF and ON state reflects on the highest absolute errors in the assessment of FF and PS phases. The lowest absolute error in the assessment of LR was reached by S-method both of OFF and ON state. The lowest absolute error in the estimation of FF and Sw was achieved by HMMspt, instead the HMMsst and HMMspt allowed for the lowest absolute error in the estimation of PS for each state. The lowest absolute error in the estimation of GPQI was achieved by HMMsst. The highest absolute error was obtained for all gait phases, both for OFF and ON state by R-method. 

The two-way repeated measure ANOVA test showed no statistical interaction for all absolute errors between Pharmacological Condition and Methods (LR: *p* = 0.71; FF: *p* = 0.16; PS: *p* = 0.71; Sw: *p* = 1.00; GPQI: *p* = 0.21). Similarly, no significant difference was found for all absolute errors between OFF and ON state (LR: *p* = 0.59; FF: *p* = 0.97; PS: *p* = 0.94; Sw: *p* = 0.26; GPQI: *p* = 0.98). Regarding the main effects of Methods, statistical difference was found for all absolute error (LR: *p* < 0.01; FF: *p* < 0.01; PS: *p* < 0.01; Sw: *p* < 0.01; GPQI: *p* < 0.01). According to the post-hoc test, statistical differences were found in the following pairwise comparisons: (i) S-method vs R-method for LR_e_ (*p* < 0.01) and Sw_e_ (*p* < 0.01; (ii) S-method vs HMMsst for LR_e_ (*p* < 0.01) and PS_e_ (*p* = 0.01); (iii) S-method vs HMMspt for LR_e_ (*p* < 0.01), FF_e_ (*p* < 0.01), PS_e_ (*p* = 0.02) and Sw_e_ (*p* = 0.01); (iv) R-method vs HMMsst for LR_e_ (*p* = 0.01), FF_e_ (*p* < 0.01), PS_e_ (*p* < 0.01), Sw_e_ (*p* < 0.01), and GPQI_e_ (*p* = 0.03); and (v) R-method vs HMMspt for FF_e_ (*p* < 0.01), PS_e_ (*p* < 0.01), Sw_e_ (*p* < 0.01), and GPQI_e_ (*p* = 0.05).

**Figure 2:**
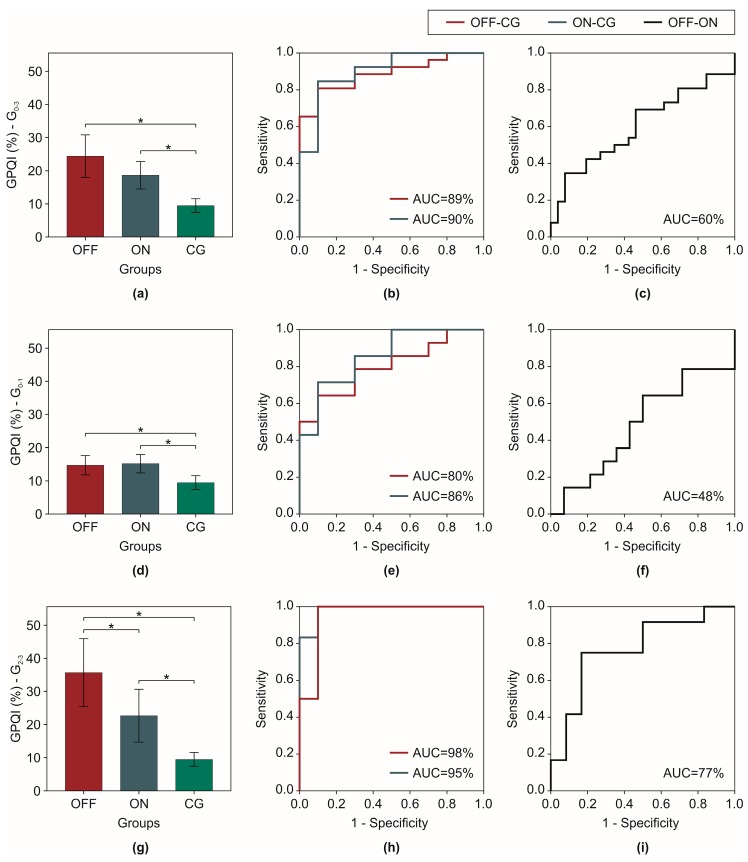
(**a**,**d**,**g**) Mean and Standard Error of GPQI of patients in both condition (OFF, ON) and control group (CG); (**b**,**e**,**h**) ROC analysis of GPQI for OFF vs CG and ON vs CG; (**c**,**f**,**i**) ROC analysis of GPQI for OFF vs ON. Each chart type is reported for three subgroups, G_0–3_, G_0–1_ and G_2–3_, respectively.

Mean and standard deviation of CG gait phases were found: (i) LR = 6.9 ± 0.7%; (ii) FF = 39.4 ± 2.3%; (iii) PS = 16.2 ± 1.8%; and (iv) Sw = 37.7 ± 0.9%. In Figure 2, mean, standard error, and ROC analysis of GPQI for patients in OFF and ON state and control groups related to G_0–3_, G_0–1_ and G_2–3_ were reported.

PD patients of all three subgroups in OFF state reported a GPQI significantly higher that CG (in G_0–3_: *p* < 0.01, in G_1–0_ *p* = 0.01, in G_2–3_ *p* < 0.01), similarly to ON state (in G_0–3_: *p* = 0.01, in G_1–0_ *p* < 0.01, in G_2–3_ *p* < 0.01). No statistical differences were found between OFF and ON state in G_0–3_ (*p* = 0.08) and G_0–1_ (*p* = 0.30), while in G_2–3_, significant difference was found between GPQI of patients in OFF and ON state (*p* = 0.03). GPQI showed a high discriminating capability in discriminating OFF parkinsonian gait from normal gait, as ROC analysis reported for all the subgroups (in G_0–3_: AUC = 89%, in G_0–3_: AUC = 80%, in G_0–3_: AUC = 98%). A similar trend was observed between PD patients in ON condition and normal gait (in G_0–3_: AUC = 90%, in G_0–3_: AUC = 86%, in G_0–3_: AUC = 95%). Instead, the accuracy of GPQI in discriminating OFF state from ON state resulted acceptable only in G_2–3_ groups (in G_0–3_: AUC = 60%, in G_0–3_: AUC = 48%, in G_0–3_: AUC = 77%).

The correlations of GPQI with clinical scales are reported in Table 5. In particular, GPQI in OFF and ON state moderately correlated with GAIT sub-item of UPDRS-III in OFF and ON state, respectively. GPQI resulted not statistically correlated with UPDRS-III score. ICC and MDC values of GPQI related to CG were 0.96 and 2.58, respectively. In Table 6, ICC and MDC values of GPQI are reported for each PD subgroup.

Regarding ICC, results showed excellent test-rest reliability both in OFF and in ON state and for each PD subgroup and in CG. The lowest value of MDC was related to the CG while the highest was related to the OFF state of PD patients with higher disease severity.

## 4. Discussion

In this study, a comparative analysis of gait partitioning methods based on four-phase model was conducted on PD patients during both OFF and ON state. Then, the validation of a novel synthetic index based on outcome parameters was performed considering a cohort of patients with Parkinson’s disease both in OFF and ON state, and a cohort of age-matched healthy subjects.

### 4.1. Technical Validity of Gait Partitioning Methods

The four-phase model, based on the estimation of LR, FF, PS and Sw phases, was analyzed by means of two threshold and two machine learning methods using a footwear-based wearable system. The comparison between gait phases obtained from tested methods and reference systems showed acceptable sensitivity and specificity rate for all methods both in OFF and ON state. However, results showed different behaviors between threshold methods. In particular, the lowest performance was achieved by R-method both in OFF and ON state. This result may be justified considering that the ruled-based filtering process, required for this methodology, could be not suitable for parkinsonian gait due to its typical abnormality. In patients with shuffling steps, the reference signals used to identify state transitions, such as cA200 and cA50, showed different shape with respect to signals related to PD without shuffling gait. Those differences in signals features led to the misdetection of state transitions of HS, TS and HO. Same results were achieved in [22] considering hemiparetic gait. Shuffling steps and jerky movements have been demonstrated to mainly limit performance of these methods, facilitating state misdetection [22]. In particular, for LR, FF, PS, and Sw of hemiparetic subjects, authors reported error values in timing estimation of 65.3, 104.1, 73.6 and 70.7 ms respectively. Our results confirmed these findings, as showed in Table 4, where R-method reported grater absolute errors. More specifically, the highest absolute error was related to flat-foot phase both in OFF and ON state due to misdetection of TS as HO. Furthermore, HO and HS events wrongly detected increase absolute errors of Sw and PS phases.

In terms of sensitivity, specificity and goodness rate, optimal results were achieved for S-method, both in OFF and ON state. Sabatini et al. [26] validated this methodology on healthy subjects reporting an average time difference of 2 ms and 35 ms in heel strike and toe-off events detection, respectively. The estimation of heel strike and toe off involving the identification of minimum peaks in foot angular velocity was already validated both in normal and PD gait [3,42]. Contrarily, the identification of toe strike and heel off events based on threshold techniques required more attention, especially in pathological gait. Our results showed that absolute value of the foot sagittal angular velocity provides reliable patterns for the assessments of those events. More specifically, the 30°/s threshold proposed in [26] provided a reliable solution in the estimation of foot stasis, typical of flat foot. Thus, this threshold method reported an optimal accuracy (G < 0.25) and a low absolute error in the estimation of gait phases and GPQI both in OFF and ON state, leading the application in parkinsonian gait monitoring.

A similar trend was achieved by machine learning approaches showing high accuracy in the estimation of gait phases, both in OFF and ON state. Authors reported HMMsst goodness index values of 0.12 ± 0.06 and 0.21 ± 0.11 for normally developed children and children with cerebral palsy [24], respectively. Whereas, 0.12 ± (0.08) and 0.21 ± (0.17) were the goodness index values reported for HMMspt [24]. Our results were similar both for HMMsst and HMMspt methods, confirming the adaptability of machine learning approach to gait patterns typical of PD. HMMsst method require patient specific training procedure, where a large amount of gait reference cycles have to be gathered, for each patient, to train the algorithm. The introduction of a standard training set related to healthy subjects avoids the training procedure, facilitating spreading of this methodology over a large population. The combination of specific pros, such as high scalability on different granularity in partitioning, applicability in daily life environments due to real-time estimation, and the great adaptability encourage the development of a wearable system based on implementation of this algorithm.

### 4.2. Clinical Validity of GPQI

Assessment of gait impairments through spatio-temporal parameters is useful both in clinical and research settings [43,44]. In the last decade, the growing development of gait monitoring systems produced a large amount of data. Thus, interpretation of results requires specific expertise, and it is often considered as time consuming. For clinical purposes, summarizing gait parameters in a synthetic index reflecting the patient’s status, would be of great impact [30].

In this study we proposed a novel synthetic index involving gait segmentation to easily assess to what extent parkinsonian gait phases distribution differs from healthy values. The GPQI index has been obtained involving reference gait phases of patient with PD both in OFF and ON state for each subgroups (G_0–3_, G_0–1_ and G_2–3_) and of age–matched healthy subjects. Results showed lower GPQI values for healthy subjects respect to patients with PD, regardless of the level of gait impairment. Furthermore, the comparison between pharmacological conditions showed significant decrease of GPQI values after pharmacological treatment for parkinsonians with severe gait abnormalities. As expected, GPQI resulted in an objective evidence to discriminate motor abnormalities between parkinsonian gait and normal condition considering the entire PD population (G_0–3_). In addition, in patients with severe gait impairments (G_2–3_), GPQI showed a capability in discriminating motor fluctuations. An increase of GPQI value reflects a worsening of patient gait conditions, allowing the discrimination between a higher gait quality induced by drug assumption, and the abnormal gait condition typical of the OFF state. The low performance of GPQI in the discrimination of pharmacological condition in G_1–0_ is due to the general mild gait involvement of these patients in OFF state, as GAIT sub–item attests. Regarding the entire patients’ population, results showed a limited capability in the discrimination between OFF and ON state, attesting that gait motor fluctuations assessment through GPQI can be useful only in patients with severe gait impairment. Parkinson’s disease, in fact, is a complex neurodegenerative disorder characterized by both motor and non-motor symptoms [45]. In some patients, gait motor impairment can be less prominent in comparison to other symptoms, such as speech dysfunctions or rest tremor. In patients with mild gait impairment, other methodologies need to be identified for monitoring of fluctuations. For the same reason, GPQI resulted significantly correlated with GAIT sub-item of UPDRS-III score in both pharmacological conditions, confirming the capability of GPQI in assessing disease severity. Otherwise, the absence of correlation between GPQI and UPDRS-III score can be justified by considering that UPDRS-III score sums a set of other sub-items, such as speech, facial expression etc., not related to gait. Furthermore, ICC values of GPQI were in the range of excellent in all cases, attesting an excellent test-retest reliability of GPQI among trials. The MDC values of GPQI were calculated for each PD subgroup in both OFF and ON state and in CG. These results encourage the use of GPQI, telling about its robustness with respect to random measurement error and inter-trial variability.

Other authors recently introduced the GVI as a standardized index for quantifying gait impairments through nine weighted spatio-temporal parameters [34]. Rennie and colleagues evaluated the validity of GVI in patients with mild to moderate PD [35]. However, GVI scores of patients with PD were found similar to GVI scores of healthy subjects, reporting low correlations between other functional performance measures. The reason of this low sensitivity of GVI in parkinsonian gait assessment was due to the inclusion of multiple spatio-temporal parameters. Key parameters become levelled out by those less important leading bias [35]. Conversely, typical gait patterns of PD patients, such as hobbling and shuffling steps, increase flat-foot and decrease swing phases. As a consequence, the GPQI score seemed more sensitive than GVI in quantifying gait quality, both in mild to moderate PD patients, and in patients with severe gait abnormalities, as supported by our results. Our preliminary findings highlighted GPQI as a useful tool for assessing levodopa effects on parkinsonian gait capability, in patients with severe gait impairments. The continuous monitoring of GPQI in real-time, made possible through machine learning approach and wearable devices, could be included into a digital diary, potentially accessible by clinicians remotely. Periodical adjustment of dopaminergic medications’ dosage could be informed by the monitoring of gait symptoms, which are crucial for some part of PD population. At the same time, if coupled with a smartphone or smartwatch app, such a device could provide feedback to patients about their current motor condition. This could be of great impact in terms of both self-awareness, a proved important factor for rehabilitation [46], and warning about risk of fall. Furthermore, several support treatments, such as rhythmic auditory stimuli proved their efficacy in improving gait performance in PD population [47]. These treatments could be triggered or modulated by means of the gait quality index, automatically evaluated in real-time.

## 5. Conclusions

Machine learning approaches showed high adaptability to different pharmacological condition. Conversely, threshold-based methods presented heterogeneous behaviors. Caution is required in choosing the suitable threshold algorithm in PD applications. Among other high-performing methods, HMMspt presents some additional benefits. This algorithm could be applied in daily monitoring in real-time. Furthermore, the inter-subjects procedure, already proposed in literature for other subjects’ populations, allows for the possibility to avoid training procedure, simplifying home monitoring applications. The novel index proposed (GPQI) demonstrated to be an objective tool to address the effects of pharmacological treatment on gait impairments in PD. The significant correlation with clinical scale items encourages its adoption in daily monitoring through wearable sensors.

## Figures and Tables

**Table 1:** Demographic data of PD patients. Patients’ motor condition is reported both in OFF and ON state as UPDRS-III score and GAIT item (0 = Normal; 1 = Mild difficulty, may not swing arms or may tend to drag leg; 2 = Moderate difficulty, requires little or no assistance; 3 = Severe disturbance of walking, requiring assistance).

Patients	Gender	Age	OFF State		ON State	
			UPDRS-III	GAIT	UPDRS-III	GAIT
1	M	68	23	0	14	0
2	M	74	25	0	10	0
3	F	71	14	0	10	0
4	M	68	32	1	19	1
5	M	66	10	1	6	0
6	F	78	26	1	23	1
7	F	73	25	1	18	1
8	F	71	15	1	12	0
9	M	78	32	1	23	1
10	M	70	18	1	8	0
11	M	74	33	1	28	1
12	F	69	27	1	20	1
13	M	64	40	1	25	0
14	F	79	36	1	26	1
15	M	81	24	2	18	1
16	M	76	33	2	22	1
17	F	51	18	2	15	2
18	M	63	38	2	20	2
19	M	77	40	2	32	2
20	M	71	42	2	31	1
21	M	74	31	2	19	1
22	M	79	30	2	19	1
23	F	71	29	2	19	1
24	F	76	43	3	20	1
25	M	75	37	3	26	2
26	M	64	36	3	28	2

**Table 2:** Brief description of the gait partitioning methods adopted in this comparative study.

	Typology	Sensor	Signals	Filters	Method Description
S-method [26]	Threshold	Single gyroscope	Sagittal angular velocity of foot	2nd order low-pass Butterworth filter with 15 Hz cut off frequency	Absolute value of the reference signal is used for identifying gait events. Starting from FS, the HO/TS time instants occurred when the absolute value of angular velocity exceed/was less than 30°/s, respectively. TO was the maximum value of the angular velocity in the clockwise direction after HO. After mid-swing, HS was identified as the second maximum value of the angular velocity in the clockwise direction.
R-method [22]	Threshold	Single 3-axes accelerometer	Radial and tangential component of foot acceleration	2nd order low-pass Butterworth filter with 6 Hz cut off frequency and two low pass moving average filters.	Gait events are identified by processing five reference signals: (i) the resultant acceleration filtered with the 2nd order low-pass Butterworth with 6 Hz cut off frequency (c50); (ii) its 1st and (iii) 2nd derivatives; and, the resultant acceleration filtered with low pass moving average filter with 1.25 s (iv) and 0.30 s (v) of window length (cA200 and cA50, respectively). The 1st and 2nd derivatives of resultant acceleration provide turning and inflection points needed to gait events identification. The two moving average filtered signals provide constraining ranges allowing the correct identification of turning and inflection points identification.
HMMsst [28]	Machine learning	Single gyroscope	Sagittal angular velocity of foot	2nd order low-pass Butterworth filter with 17 Hz cut off frequency	This method processes the sagittal angular velocity of the foot, based on the scalar continuous Hidden Model Markov (cHMM). Gait phases are obtained as the likely sequence of the hidden states. The starting point is the training procedure involving the Baum-Welch algorithm and a set of model parameters: (i) the probability distribution matrix of the transition state, chosen as a left-right model; (ii) the initial state vector distribution, chosen giving the same probability for all phases, (iii) a vector of mixture coefficients, i.e., the weights used to estimate the sequence of states; and (iv) the mean and the standard deviation of the signal. This algorithm is trained with signals gathered from two trials of a patient in a specific pharmacological condition (OFF or ON), and tested with leaved out trial of the same patient.
HMMspt [24]	Machine learning	Single gyroscope	Sagittal angular velocity of foot	2nd order low-pass Butterworth filter with 17 Hz cut off frequency	Similar to the previous method, a scalar continuous Hidden Model Markov is used to estimate gait sequence as the likely sequence. For patients with motor deficit, the training procedure involves mean and standard deviation of foot sagittal angular velocity of all trials of control group. Afterwards, foot sagittal angular velocity of all trials of patients is tested to estimate likely gait sequence.

**Table 3:** Mean and standard deviation of True Positive Rate (TPR), True Negative Rate (TNR) and Goodness index (G) of all methods both in OFF and ON state of right side.

	OFF State				ON State			
	S-Method	R-Method	HMMsst	HMMspt	S-Method	R-Method	HMMsst	HMMspt
TPR	0.9 (0.1)	0.8 (0.2)	0.9 (0.1)	0.9 (0.1)	1.0 (0.1)	0.8 (0.1)	1.0 (0.1)	0.9 (0.1)
TNR	0.9 (0.0)	0.7 (0.1)	0.9 (0.1)	0.9 (0.0)	0.9 (0.1)	0.7 (0.1)	0.9 (0.1)	0.9 (0.0)
G	0.1 (0.1)	0.4 (0.2)	0.1 (0.1)	0.1 (0.1)	0.1 (0.1)	0.4 (0.2)	0.1 (0.1)	0.1 (0.1)

**Table 4:** Mean and Standard Deviation of absolute error in the estimation: Loading Response (LR), Flat-Foot (FF), Pre-Swing (PS), Swing (Sw) and GPQI both for OFF and ON state.

	OFF State				ON State			
	S-Method	R-Method	HMMsst	HMMspt	S-Method	R-Method	HMMsst	HMMspt
LR_e_ (%)	0.7 (1.2)	4.7 (4.2)	2.5 (1.8)	3.8 (2.0)	0.5 (0.6)	5.4 (5.1)	2.6 (1.8)	3.7 (2.0)
FF_e_ (%)	5.4 (3.5)	10.4 (7.9)	3.9 (3.0)	2.8 (2.2)	6.0 (4.3)	11.7 (11.6)	3.8 (2.8)	3.0 (2.0)
PS_e_ (%)	5.9 (3.5)	9.2 (5.6)	3.2 (2.3)	3.3 (3.9)	6.2 (4.6)	9.6 (6.2)	3.5 (3.0)	2.8 (2.9)
Sw_e_ (%)	2.3 (2.6)	8.0 (5.1)	2.4 (1.8)	1.6 (1.8)	1.8 (1.7)	9.1 (6.5)	2.1 (1.4)	1.1 (0.9)
GPQI_e_ (%)	4.7 (3.8)	10.0 (12.0)	3.2 (3.9)	3.2 (2.3)	5.6 (3.6)	10.1 (13.1)	3.7 (4.4)	4.1 (4.1)

**Table 5:** *p*-Values and correlation coefficient of Spearman’s Rho analysis of GPQI for the entire parkinsonian population. Statistical significant differences are starred.

	OFF State		ON State	
	UPDRS-III	GAIT	UPDRS-III	GAIT
GPQI	*p* = 0.09 r = 0.33	*p* < 0.01 * r = 0.60	*p* = 0.17 r = 0.30	*p* = 0.03 * r = 0.43

**Table 6:** ICC values and MDC values of GPQI index for the three subgroups, G_0–3_, G_0–1_ and G_2–3_.

	OFF State		ON State	
	ICC_3,k_	MDC_95%_	ICC_3,k_	MDC_95%_
G_0–3_	0.99	4.87	0.99	3.78
G_0–1_	0.97	3.28	0.98	2.89
G_2–3_	0.99	5.95	0.99	4.50

## References

[1] Taborri J., Palermo E., Rossi S., Cappa P. (2016). Gait Partitioning Methods: A Systematic Review. Sensors.

[2] Hegde N., Bries M., Sazonov E. (2016). A Comparative Review of Footwear-Based Wearable Systems. Electronics.

[3] Salarian A., Russmann H., Vingerhoets F.J.G., Dehollain C., Blanc Y., Burkhard P.R., Aminian K. (2004). Gait assessment in Parkinson’s disease: Toward an ambulatory system for long-term monitoring. IEEE Trans. Biomed. Eng..

[4] Mileti I., Taborri J., Rossi S., Petrarca M., Patane F., Cappa P. Evaluation of the effects on stride-to-stride variability and gait asymmetry in children with Cerebral Palsy wearing the WAKE-up ankle module. Proceedings of the 2016 IEEE International Symposium on Medical Measurements and Applications (MeMeA).

[5] Hundza S., Hook W., Harris C., Mahajan S., Leslie P., Spani C., Spalteholz L., Birch B., Commandeur D., Livingston N. (2013). Accurate and Reliable Gait Cycle Detection in Parkinson’s Disease. IEEE Trans. Neural Syst. Rehabil. Eng..

[6] Patel S., Lorincz K., Hughes R., Huggins N., Growdon J., Standaert D., Akay M., Dy J., Welsh M., Bonato P. (2009). Monitoring motor fluctuations in patients with Parkinson’s disease using wearable sensors. IEEE Trans. Inf. Technol. Biomed..

[7] Kestenbaum M., Fahn S. (2015). Safety of IPX066, an extended release carbidopa-levodopa formulation, for the treatment of Parkinson’s disease. Expert Opin. Drug Saf..

[8] Jankovic J. (2008). Parkinson’s disease: Clinical features and diagnosis. J. Neurol. Neurosurg. Psychiatry.

[9] Zesiewicz T.A., Sullivan K.L., Hauser R.A. (2007). Levodopa-induced dyskinesia in Parkinson’s disease: Epidemiology, etiology, and treatment. Curr. Neurol. Neurosci. Rep..

[10] Thanvi B., Lo N., Robinson T. (2007). Levodopa-induced dyskinesia in Parkinson’s disease: Clinical features, pathogenesis, prevention and treatment. Postgrad. Med. J..

[11] Movement Disorder Society Task Force on Rating Scales for Parkinson’s Movement Disorder Society Task Force on Rating Scales for Parkinson’s Disease (2003). The Unified Parkinson’s Disease Rating Scale (UPDRS): Status and recommendations. Mov. Disord..

[12] Haji Ghassemi N., Hannink J., Martindale C., Gaßner H., Müller M., Klucken J., Eskofier B. (2018). Segmentation of Gait Sequences in Sensor-Based Movement Analysis: A Comparison of Methods in Parkinson’s Disease. Sensors.

[13] Hausdorff J.M., Lowenthal J., Herman T., Gruendlinger L., Peretz C., Giladi N. (2007). Rhythmic auditory stimulation modulates gait variability in Parkinson’s disease. Eur. J. Neurosci..

[14] Hausdorff J.M., Rios D.A., Edelberg H.K. (2001). Gait variability and fall risk in community-living older adults: A 1-year prospective study. Arch. Phys. Med. Rehabil..

[15] Plotnik M., Giladi N., Balash Y., Peretz C., Hausdorff J.M. (2005). Is freezing of gait in Parkinson’s disease related to asymmetric motor function?. Ann. Neurol..

[16] Mariani B., Rouhani H., Crevoisier X., Aminian K. (2013). Quantitative estimation of foot-flat and stance phase of gait using foot-worn inertial sensors. Gait Posture.

[17] Zhu H.S., Wertsch J.J., Harris G.F., Loftsgaarden J.D., Price M.B. (1991). Foot pressure distribution during walking and shuffling. Arch. Phys. Med. Rehabil..

[18] Pacilli A., Mileti I., Germanotta M., Di Sipio E., Imbimbo I., Aprile I., Padua L., Rossi S., Palermo E., Cappa P. A wearable setup for auditory cued gait analysis in patients with Parkinson’s Disease. Proceedings of the 2016 IEEE International Symposium on Medical Measurements and Applications (MeMeA).

[19] Hughes J., Bowes S., Leeman A., O’Neill C., Deshmukh A., Nicholson P., Dobbs S., Dobbs R. (1990). Parkinsonian abnormality of foot strike: A phenomenon of ageing and/or one responsive to levodopa therapy?. Br. J. Clin. Pharmacol..

[20] Nieuwboer A., De Weerdt W., Peeraer L., Lesaffre E., Hilde F., Baunach B. (1999). Plantar force distribution in Parkinsonian gait: A comparison between patients and age-matched control subjects. Scand. J. Rehabil. Med..

[21] Koller W.C., Glatt S., Vetere-Overfield B., Hassanein R. (1989). Falls and Parkinson’s disease. Clin. Neuropharmacol..

[22] Rueterbories J., Spaich E.G., Andersen O.K. (2014). Gait event detection for use in FES rehabilitation by radial and tangential foot accelerations. Med. Eng. Phys..

[23] Mannini A., Sabatini A.M. (2012). Gait phase detection and discrimination between walking–jogging activities using hidden Markov models applied to foot motion data from a gyroscope. Gait Posture.

[24] Taborri J., Scalona E., Palermo E., Rossi S., Cappa P. (2015). Validation of inter-subject training for hidden markov models applied to gait phase detection in children with Cerebral Palsy. Sensors.

[25] Taborri J., Scalona E., Rossi S., Palermo E., Patanè F., Cappa P. Real-time gait detection based on Hidden Markov Model: Is it possible to avoid training procedure?. Proceedings of the 2015 IEEE International Symposium on Medical Measurements and Applications (MeMeA).

[26] Sabatini A.M., Martelloni C., Scapellato S., Cavallo F. (2005). Assessment of walking features from foot inertial sensing. IEEE Trans. Biomed. Eng..

[27] Mannini A., Trojaniello D., Della Croce U., Sabatini A.M. Hidden Markov model-based strategy for gait segmentation using inertial sensors: Application to elderly, hemiparetic patients and Huntington’s disease patients. Proceedings of the 2015 37th Annual International Conference of the IEEE Engineering in Medicine and Biology Society (EMBC).

[28] Taborri J., Rossi S., Palermo E., Patanè F., Cappa P. (2014). A Novel HMM Distributed Classifier for the Detection of Gait Phases by Means of a Wearable Inertial Sensor Network. Sensors.

[29] Galli M., Cimolin V., De Pandis M.F., Schwartz M.H., Albertini G. (2012). Use of the Gait Deviation Index for the Evaluation of Patients With Parkinson’s Disease. J. Mot. Behav..

[30] Schutte L.M., Narayanan U., Stout J.L., Selber P., Gage J.R., Schwartz M.H. (2000). An index for quantifying deviations from normal gait. Gait Posture.

[31] Malt M.A., Aarli Å., Bogen B., Fevang J.M. (2016). Correlation between the Gait Deviation Index and gross motor function (GMFCS level) in children with cerebral palsy. J. Child. Orthop..

[32] Baker R., McGinley J.L., Schwartz M.H., Beynon S., Rozumalski A., Graham H.K., Tirosh O. (2009). The Gait Profile Score and Movement Analysis Profile. Gait Posture.

[33] Gouelle A., Mégrot F., Presedo A., Husson I., Yelnik A., Penneçot G.-F. (2013). The Gait Variability Index: A new way to quantify fluctuation magnitude of spatiotemporal parameters during gait. Gait Posture.

[34] Balasubramanian C.K., Clark D.J., Gouelle A. (2015). Validity of the Gait Variability Index in older adults: Effect of aging and mobility impairments. Gait Posture.

[35] Rennie L., Dietrichs E., Moe-Nilssen R., Opheim A., Franzén E. (2017). The validity of the Gait Variability Index for individuals with mild to moderate Parkinson’s disease. Gait Posture.

[36] Gelb D.J., Oliver E., Gilman S. (1999). Diagnostic criteria for Parkinson disease. Arch. Neurol..

[37] Tests D. (1978). Basic Principles of ROC Analysis. Semin. Nucl. Med..

[38] Dancey C.P., Reidy J. (2007). Statistics without Maths for Psychology: Using SPSS for Windows.

[39] Shrout P.E., Fleiss J.L. (1979). Intraclass correlations: Uses in assessing rater reliability. Psychol. Bull..

[40] Cicchetti D.V. (1994). Guidelines, criteria, and rules of thumb for evaluating normed and standardized assessment instruments in psychology. Psychol. Assess..

[41] Di Fabio R.P. (2013). Essentials of Rehabilitation Research: A Statistical Guide to Clinical Practice.

[42] Mileti I., Germanotta M., Alcaro S., Pacilli A., Imbimbo I., Petracca M., Erra C., Di Sipio E., Aprile I., Rossi S. Gait partitioning methods in Parkinson’s disease patients with motor fluctuations: A comparative analysis. Proceedings of the 2017 IEEE International Symposium on Medical Measurements and Applications (MeMeA).

[43] Motta C., Palermo E., Studer V., Germanotta M., Germani G., Centonze D., Cappa P., Rossi S., Rossi S. (2016). Disability and Fatigue Can Be Objectively Measured in Multiple Sclerosis. PLoS ONE.

[44] Casamassima F., Ferrari A., Milosevic B., Ginis P., Farella E., Rocchi L. (2014). A Wearable System for Gait Training in Subjects with Parkinson’s Disease. Sensors.

[45] Magrinelli F., Picelli A., Tocco P., Federico A., Roncari L., Smania N., Zanette G., Tamburin S. (2016). Pathophysiology of Motor Dysfunction in Parkinson’s Disease as the Rationale for Drug Treatment and Rehabilitation. Park. Dis..

[46] Medley A.R., Powell T. (2010). Motivational Interviewing to promote self-awareness and engagement in rehabilitation following acquired brain injury: A conceptual review. Neuropsychol. Rehabil..

[47] Thaut M.H., Abiru M. (2010). Rhythmic Auditory Stimulation in Rehabilitation of Movement Disorders: A Review of Current Research. Music Percept..

